# Depressive Symptoms in Pediatric Tuberculosis: A Retrospective Two-Time-Point Observational Study

**DOI:** 10.3390/diseases14050157

**Published:** 2026-04-29

**Authors:** Oana Mariana Mihailov, Loredana Stavăr Matei, George Țocu, Valerii Luțenco, Cosmin George Popovici, Raul Mihailov

**Affiliations:** 1 Faculty of Medicine and Pharmacy, Research Center in the Medical-Pharmaceutical Field, “Dunarea de Jos” University, 800008 Galati, Romania; oana.mihailov@ugal.ro (O.M.M.); loredana.matei@ugal.ro (L.S.M.); valerii.lutenco@ugal.ro (V.L.); dr.popovicigeorgecosmin@gmail.com (C.G.P.); raul.mihailov@ugal.ro (R.M.); 2 Pneumophthisiology Hospital “Sfântul Spiridon”, 800189 Galati, Romania

**Keywords:** pediatric tuberculosis, depressive symptoms, psychological screening, children’s mental health, retrospective study, inflammation, chronic disease

## Abstract

Background: Tuberculosis (TB) in children is associated not only with infectious burden but also with potential psychological distress, which remains insufficiently explored. The aim of this study was to evaluate the pattern and evolution of depressive symptoms in pediatric TB patients during treatment using a structured screening approach. Methods: We conducted a retrospective observational study including 190 pediatric patients aged 7–18 years diagnosed with tuberculosis between 2019 and 2021. Depressive symptoms were assessed at two time points, namely at diagnosis (T0) and at first follow-up (T1), using a 10-item structured clinical screening tool routinely applied in practice. A threshold of ≥50% affirmative responses was used to identify patients with suspected depressive symptoms. The Children’s Depression Inventory (CDI) was administered to patients with positive screening results, according to standard clinical protocols. Descriptive and comparative analyses were performed to evaluate changes over time. Results: A high proportion of patients screened positive for depressive symptoms at baseline (T0). At follow-up (T1), a reduction in the proportion of patients with suspected depressive symptoms was observed; however, a substantial number of patients continued to report symptoms suggestive of emotional distress. Most symptom changes between T0 and T1 were not statistically significant, with the exception of decreased appetite, which showed a modest improvement. The overall pattern suggests persistence of symptoms in a subset of patients over time. Conclusions: These findings suggest that symptoms indicative of psychological distress are common among pediatric TB patients and may persist during treatment. However, given the use of a non-validated screening tool and the retrospective design, the results should be interpreted with caution. The study highlights the potential value of systematic psychological assessment in this population and supports the need for further research using validated instruments.

## 1. Introduction

Tuberculosis (TB) remains a major global health concern, affecting millions of individuals each year despite advances in diagnosis and treatment [1,2]. According to the World Health Organization, children represent a particularly vulnerable population, both in terms of disease burden and diagnostic challenges [1]. In Eastern Europe, and particularly in Romania, pediatric TB incidence remains higher compared to many other European countries [2], emphasizing the need for a comprehensive approach to care.

The interaction between tuberculosis and mental health has been increasingly recognized in recent years. Studies conducted in adult populations have consistently shown a high proportion of screened patients with depressive symptoms among TB patients, with important implications for treatment adherence and clinical outcomes [3,4]. For instance, Sweetland et al. described tuberculosis and depression as a syndemic, linked through interacting biological, behavioral, and psychosocial mechanisms that may worsen disease burden and treatment outcomes [5].

In pediatric populations, however, the psychological dimension of TB remains insufficiently explored. Emotional distress in children is often under-recognized, as somatic symptoms tend to dominate the clinical presentation [6]. This may delay appropriate psychological intervention and contribute to poorer outcomes. Furthermore, stigma, prolonged treatment, and hospitalization may increase psychological vulnerability, particularly among adolescents [7].

Romania continues to report the highest TB incidence rates in the European Union, with pediatric cases contributing significantly to the national burden of the disease [8].

Despite this epidemiological context, there is a lack of studies systematically evaluating the prevalence and evolution of depressive symptoms in children with TB in this setting.

Given these considerations, the present study aims to evaluate the prevalence and longitudinal evolution of depressive symptoms in pediatric TB patients using a structured screening approach applied at two defined time points during treatment.

TB, one of the oldest known infectious diseases, continues to represent a major global public health challenge [9,10]. Children and adolescents are particularly vulnerable to emotional distress due to factors such as prolonged hospitalization, social isolation, stigma, treatment fatigue, family stress, and school interruption [11].

Moreover, depression-related non-adherence has been linked to therapeutic failure, development of antimicrobial resistance, and increased mortality [12,13]. Similar findings have been reported in international reports; for example, a UK–Pakistan study found that depression, anxiety, and negative illness perceptions were independently associated with poor treatment adherence in TB patients [14]. In children, depressive symptoms are frequently unrecognized because physical illness often masks emotional distress, leading to delayed or absent psychological interventions.

This is further supported by findings from Ambaw et al. [4], who prospectively demonstrated that untreated or persistent depressive symptoms can negatively influence TB treatment pathways and adherence.

Current data regarding the prevalence and evolution of depressive symptoms in children with TB are limited, especially in Eastern European settings. To our knowledge, no previous study in Romania has systematically evaluated the dynamics of depressive symptoms in pediatric TB patients during treatment.

In this context, the present study aims to assess the pattern and evolution of depressive symptoms in children diagnosed with tuberculosis, using a structured psychological screening approach at two defined time points during treatment.

## 2. Materials and Methods

### 2.1. Study Design and Setting

This retrospective observational study was conducted within the Pediatric Pneumology Section of the “Sf. Spiridon” Pneumophthisiology Hospital, Galați, Romania, and affiliated outpatient TB dispensaries in Galați, Tecuci, and Târgu Bujor, Galați County, Romania. These centers operate under the framework of the Romanian National Tuberculosis Control Program (Romanian NTP) and follow standardized diagnostic and treatment protocols aligned with World Health Organization (WHO) guidelines.

The inclusion of both hospital-based and outpatient cases was intended to provide a more comprehensive representation of pediatric TB patients across different levels of care.

### 2.2. Participants and Eligibility Criteria

The study included pediatric patients aged 7–18 years diagnosed with tuberculosis between 2019 and 2021. Eligible participants had complete medical records and documented psychological screening at both predefined time points.

Patients were excluded if medical records were incomplete or if psychological evaluation data were unavailable at either assessment point.

### 2.3. Data Sources and Collection

Data were extracted from routinely completed clinical and psychological records, including inpatient observation sheets and outpatient follow-up documentation.

Of the total study population (*n* = 190), 85 patients were evaluated in the hospital setting, while 105 cases were managed in outpatient TB dispensaries (Galați: 40; Tecuci: 45; Târgu Bujor: 20).

The cohort included patients managed both in the hospital setting and in outpatient TB dispensaries. Of the total study population (*n* = 190), the majority were evaluated within the hospital setting, while the remaining cases were followed in outpatient facilities in Galați, Tecuci, and Târgu Bujor.

### 2.4. Clinical Setting and Patient Pathway

In Romania, pediatric TB patients are diagnosed, treated, and monitored according to national protocols integrated within the NTB Registry. Management follows standardized regimens consistent with international recommendations, ensuring continuity of care between hospital admission and outpatient follow-up.

### 2.5. Psychological Assessment Protocol

Psychological evaluation was performed at two predefined time points: at diagnosis/initiation of treatment (T0) and at the first scheduled follow-up visit (T1).

A structured 10-item screening interview was used as part of a routine clinical psychological assessment. The items were selected to capture common emotional and behavioral features associated with depressive symptomatology in pediatric populations, including mood changes, fatigue, sleep disturbances, appetite changes, irritability, and psychosocial stressors. Each item was scored dichotomously (yes/no).

A threshold of ≥50% affirmative responses was applied as a pragmatic clinical criterion to identify patients with suspected depressive symptoms. This threshold was used in routine practice to prioritize sensitivity in detecting children at potential psychological risk and was not intended as a diagnostic cutoff.

Given that the screening tool was not formally validated, it should be interpreted as an initial risk assessment rather than a diagnostic instrument. To improve specificity and support clinical interpretation, patients who screened positive at either time point were further evaluated using the Children’s Depression Inventory (CDI).

The CDI is a widely validated instrument for assessing depressive symptoms in children and adolescents, demonstrating good reliability and internal consistency (Cronbach’s alpha typically >0.80), as well as satisfactory sensitivity and specificity in previous studies [15,16].

### 2.6. Statistical Analysis

Descriptive and comparative statistical analyses were performed to evaluate the distribution and evolution of depressive symptoms between T0 and T1.

Continuous variables were summarized using mean, median, and standard deviation, while categorical variables were expressed as frequencies and percentages. Comparisons between groups were performed using appropriate statistical tests (chi-square or Fisher’s exact test, as applicable). Pearson correlation coefficients were used to assess associations between screening results and CDI scores. A *p*-value < 0.05 was considered statistically significant. All statistical analyses were performed using IBM SPSS Statistics, version 26.0 (IBM Corp., Armonk, NY, USA).

## 3. Results

### 3.1. Socio-Demographic Characteristics

A total of 190 pediatric patients diagnosed with tuberculosis were included in the study. The majority of patients were from rural areas (*n* = 134, 70.5%), while 56 (29.5%) were from urban settings. Adolescents (15–18 years) represented the largest age group.

The distribution of clinical forms showed a predominance of occult primary tuberculosis (*n* = 155, 81.6%), followed by secondary tuberculosis (*n* = 21, 11.1%), primary tuberculosis with adenopathy (*n* = 10, 5.3%), and tuberculous pleurisy (*n* = 4, 2.1%). Detailed sociodemographic characteristics are presented in Table 1.

The most frequent clinical form was occult primary tuberculosis (81.6%), followed by secondary tuberculosis (11.1%), primary tuberculosis with adenopathy (5.3%), and tuberculous pleurisy (2.1%).

### 3.2. Prevalence of Suspected Depressive Symptoms at T0 and T1

At baseline (T0), 171 out of 190 patients (90.0%) met the screening threshold for suspected depressive symptoms, while 19 (10.0%) did not.

At follow-up (T1), the proportion of patients with suspected depressive symptoms decreased to 141 (74.2%), whereas 39 patients (20.5%) no longer met the screening criteria. Follow-up data were unavailable for 10 patients (5.3%). A comparative summary of screening outcomes at T0 and T1 is presented in Table 2.

### 3.3. Symptom-Level Analysis

Changes in individual screening items between T0 and T1 are presented in Table 3.

Among the evaluated symptoms, only decreased appetite showed a statistically significant difference over time (*p* = 0.034). All other symptoms demonstrated non-significant variations, indicating relative stability of most psychological features during the observation period.

### 3.4. CDI Assessment

The Children’s Depression Inventory (CDI) was administered to 158 patients (83.2%), corresponding to the majority of children who screened positive at either assessment time point. The instrument was not applied in 32 cases (16.8%), primarily due to absence at follow-up or incomplete screening data.

### 3.5. Correlation Analysis

Pearson correlation analysis between screening items and CDI scores is presented in Table 4.

At T0, several variables showed significant correlations with CDI scores, including personal or family problems, irritability, fatigue, insomnia, decreased appetite, low self-esteem, substance use, and introversion.

At T1, significant correlations were observed for inefficiency, insomnia, fatigue, personal or family problems, introversion, irritability, and low self-esteem, while decreased appetite showed a borderline association.

### 3.6. CDI Results

The results of the decision to administer the Children’s Depression Inventory (CDI) are shown in Table 5.

The Children’s Depression Inventory was administered to the majority of patients with positive screening results. The analysis showed a consistent association between screening findings and CDI scores, supporting the validity of the screening approach.

Correlation patterns differed between the two time points, suggesting a dynamic evolution of depressive symptom expression during treatment.

## 4. Discussion

The present study highlights a high proportion of screened patients with depressive symptoms among pediatric patients diagnosed with tuberculosis [4,5], both at baseline and during follow-up. These findings are consistent with previous research conducted in adult populations, where TB has been associated with increased psychological vulnerability, influenced by both biological and psychosocial factors [4,17].

The predominance of patients from rural areas in our cohort may contribute to the observed psychological burden. Previous studies suggest that rural residence is often associated with delayed access to healthcare services [18], increased social vulnerability, and limited availability of specialized mental health support. In this context, the high proportion of adolescents in our study group may further explain the elevated rate of depressive symptoms at baseline, given the known emotional sensitivity of this developmental stage.

The persistence of depressive symptoms at T1, despite a modest reduction compared to baseline, suggests that emotional distress does not resolve spontaneously with clinical improvement. This observation supports the hypothesis that psychological burden in pediatric TB patients follows a more prolonged course and may require targeted interventions beyond standard medical treatment.

Although our study did not directly assess causal mechanisms, several factors may contribute to this pattern, including prolonged hospitalization, social isolation, stigma, and uncertainty related to disease evolution. These aspects have been previously described in the literature as important contributors to psychological distress in TB patients.

Interestingly, while a slight decrease in the number of reported symptoms was observed over time, the overall pattern remained relatively stable. This finding suggests that, in the absence of structured psychological support, depressive symptoms may persist throughout treatment. Similar observations have been reported in other chronic pediatric conditions, where emotional adaptation is often incomplete without targeted intervention.

The analysis of symptom patterns also revealed variability in individual trajectories, indicating that psychological responses to TB are heterogeneous. While some children showed improvement, others maintained high symptom scores, highlighting the need for individualized monitoring and intervention strategies.

The use of the Children’s Depression Inventory (CDI) supported the validity of the screening approach, as previously demonstrated in pediatric populations. The correlations observed between screening items and CDI scores further reinforce the relevance of routine psychological assessment in clinical practice.

Taken together, these findings emphasize the importance of integrating mental health evaluation into standard TB care for children. Early identification of at-risk patients may facilitate timely intervention, potentially improving both psychological well-being and treatment outcomes.

Similar increases in anxiodepressive symptom burden under conditions of prolonged stress and uncertainty have been reported in pediatric and young populations during major societal stressors, supporting the role of contextual and environmental factors in symptom persistence [19]. The persistence of suspected depressive symptoms at T1, although at a lower level than at T0, suggests that emotional distress remains relevant throughout treatment and does not completely resolve after clinical stabilization. Additionally, recent evidence shows that depression may negatively influence TB outcomes through biological pathways as well [20,21,22]. Liu et al. showed that TB patients with depressive or anxiety symptoms had impaired cellular immune status and stronger inflammatory responses, suggesting that these psycho-emotional disturbances may adversely affect mechanisms relevant to TB control and clinical recovery [23]. These findings are further supported by Mocanu et al. [24], who describe behavioral and neuroimmune mechanisms through which depression can worsen TB progression and treatment response, reinforcing the bidirectional relationship between psychological distress and disease severity. Similar associations between inflammatory biomarkers and adverse clinical outcomes have been reported in other infectious conditions. For example, elevated procalcitonin levels have been shown to predict 28-day mortality in patients with surgical sepsis and septic shock, as demonstrated by Tocu et al. [25]. Although TB follows a different immunopathogenic pathway, these findings highlight the broader relevance of biological predictors in understanding disease severity and patient prognosis. Taken together, these data highlight the necessity of continuous psychological monitoring throughout treatment, rather than relying on a single-point assessment.

The predominance of occult primary tuberculosis (81.6%) is noteworthy, as primary TB often requires prolonged hospitalization and repeated clinical evaluations, factors that have been shown to increase emotional distress in pediatric patients. Although our analysis did not stratify depressive symptoms by TB subtype, the clinical burden associated with more complex presentations, such as secondary TB or pleurisy, may contribute to heightened psychological vulnerability and should be explored in future research.

The descriptive statistics of total symptom counts revealed only a modest decline in the mean number of affirmative items from T0 (mean = 5.85) to T1 (mean = 5.30). The relatively small reduction and the overlapping score distributions underscore the chronicity and persistence of depressive symptoms in pediatric TB patients. Interestingly, the minimum score decreased to 0 at T1, indicating that a minority of children experienced complete remission of emotional symptoms, whereas others continued to score as high as 9 items, demonstrating substantial heterogeneity in psychological recovery.

The item-level analysis provided further nuance to these findings. Several symptoms—such as irritability, introversion, and inefficiency—showed moderate fluctuations between T0 and T1 but without statistical significance. This pattern suggests that while some children may experience an episodic improvement in certain domains, the underlying psychological burden remains largely unchanged. Particularly notable is the marked, though non-significant, reduction in irritability (from 84.2% to 45%), which may reflect partial emotional adjustment following treatment initiation.

Comparison of results between T0 and T1 showed that most symptoms remained relatively stable over time, with only decreased appetite showing a statistically significant reduction. Although this is a somatic rather than cognitive symptom, its improvement may reflect partial adaptation to treatment and reduced physical discomfort. While a lower proportion of children screened positive for depressive symptoms at T1 (74.2%) compared to T0 (90%), most symptom differences between the two assessment points were not statistically significant, with the exception of decreased appetite. This indicates that emotional distress remained highly prevalent throughout the treatment. The minimal changes observed for the other items suggest that psychological symptoms may require structured interventions beyond standard medical care. Our findings align with the concept of the TB–depression syndemic described by Sweetland et al. [5], which highlights how tuberculosis and depression exacerbate one another and underscores the importance of integrating mental health services into TB programs.

The CDI results further support the relevance of the screening protocol. The instrument was administered to the majority of children who screened positive at any of the assessment time points, and correlation analysis confirms that many emotional and behavioral symptoms identified in routine screening correspond well with standardized measures of depressive severity. At T0, the strongest association was observed for problems in personal or family life, highlighting the major impact of the family context on the child’s emotional state at the time of diagnosis. At T1, inefficiency, insomnia, and fatigue had the strongest correlations with CDI scores, suggesting that functional impairments and somatic symptoms may persist as important indicators of depressive burden as treatment progresses.

The high rate of CDI administration (83.2%) also reflects the substantial psychological concern observed in clinical practice. The 16.8% of cases where CDI was not administered correspond mainly to absent follow-up or insufficient screening results, underscoring the need for improved continuity of psychological evaluation within TB programs.

The correlation matrix revealed a distinct pattern of associations across the two time points. At T0, the exceptionally high correlation between family/personal problems and CDI scores (r = 0.783) highlights the central role of familial and environmental stressors at the time of diagnosis. By contrast, at T1, inefficiency emerged as the strongest predictor of CDI severity (r = 0.421), suggesting that functional difficulties—rather than purely emotional symptoms—may become more salient as treatment progresses. This temporal shift underscores the dynamic nature of depressive manifestations in pediatric TB.

Taken together, the descriptive, comparative, and correlational analyses reveal a consistent pattern: depressive symptoms in pediatric TB patients are both highly prevalent and persistent, with only minimal natural remission over time. The data emphasize the importance of early detection, multidimensional evaluation, and sustained psychological support throughout the course of TB treatment.

These findings demonstrate that multi-stage screening is effective in identifying children at increased psychological risk, and the CDI provides valuable additional information for clinical assessment. Targeted psychological interventions aimed at improving treatment acceptance, such as motivational interviewing, have been associated with improved adherence, symptom reduction, and better patient engagement in adolescents and young adults with chronic medical conditions, suggesting potential benefits if incorporated into pediatric TB care [26,27]. The results highlight the importance of integrating mental health support into TB management, especially given that depressive symptoms can influence treatment adherence, quality of life, and overall recovery.

The study also addresses a significant gap in pediatric TB research in Romania, as there have been no studies to date that systematically assess depressive symptoms in this population. By documenting symptom patterns at two time points and validating the screening protocol in relation to CDI scores, the study provides a basis for implementing structured psychosocial monitoring within the national TB program.

Another study conducted in Butajira indicated a similarly high prevalence (54%) of depression among newly diagnosed TB patients [4]. Kam A et al., in their study conducted in Toronto, Canada, which aimed to intersect TB and mental health, highlighted that 5 adolescents out of 23, i.e., 22%, developed depressive mood symptoms during treatment and were referred to a psychiatric care service [28]. According to the study conducted by Avdeeva et al. in Smolensk, Russia, 61% of adolescents undergoing TB treatment experienced anxiety (proven using Taylor’s Manifest Anxiety Scale) compared to 35% of healthy adolescents without TB [29,30].

Wang et al. [31] highlighted the strong association between tuberculosis and depressive symptoms, driven by both biological and psychosocial mechanisms impacting treatment outcomes. Consistent with these findings, our study identified a high prevalence of depressive symptoms among pediatric TB patients at baseline (T0), followed by a reduction of 15.8% at the first follow-up (T1). Despite this improvement, the persistence of symptoms in a subset of patients supports the concept of a TB–depression syndemic [31], underlining the importance of early screening and continuous psychological assessment to optimize both mental health and treatment outcomes.

Approximately 70% of patients with probable depression also had significant anxiety symptoms, and vice versa, and 69.9% of patients with anxiety were also diagnosed with probable depression.

In pediatric tuberculosis, several sociodemographic and clinical determinants have been associated with an increased risk of depressive disorders, including rural residence, family-related stressors, and limited access to psychosocial support [32].

Since depressive and anxiety symptoms are frequently encountered among TB patients, screening for depression and anxiety can help identify patients who need additional psychosocial evaluation, support, and treatment to experience a better clinical response to anti-TB treatment.

Our findings are consistent with previous research indicating that depressive symptoms may negatively influence treatment adherence and perceived recovery among TB patients. Similar studies conducted in Ethiopia, India, and Romania [33,34,35,36,37] have reported that early psychological support and patient-centered counseling were associated with improved adherence and treatment completion rates. Although our study did not directly assess treatment outcomes, the observed reduction in depressive risk from T_0_ to T_1_ may indirectly reflect the positive effects of integrated clinical and psychological care provided during TB treatment.

TB is a global, life-threatening infection. Besides the high global prevalence of TB, it is also associated with various comorbidities. One of the most common and lethal comorbidities is depression [37]. In order to effectively prevent the onset of depressive disorders among TB patients, it is essential to examine the factors contributing to depression by evaluating their cumulative impact. While such analysis may not yield exact protocols for designing targeted prevention programs, it can offer valuable insights and cautionary guidance for healthcare providers involved in the management of TB patients [37]. Findings from internationally published research suggest that susceptibility to depressive disorders among individuals diagnosed with tuberculosis is influenced by a combination of social, educational, and treatment-related characteristics. Gender differences, restricted social resources, lower levels of formal education, residence in non-urban areas, and previous exposure to anti-tuberculosis therapy have all been reported as contributing elements across multiple epidemiological contexts during the last decade [37].

Consistent patterns have also been observed in sub-Saharan African settings. In an Ethiopian cohort comprising 409 patients with tuberculosis, a considerable proportion had limited educational attainment and lived in rural environments. Within this population, insufficient social support was strongly associated with depressive symptomatology, with affected individuals demonstrating a substantially higher probability of emotional distress compared with those reporting moderate or robust support networks [33]. A meta-analysis comprising eight studies identified a strong association between depressive symptoms and unfavorable TB treatment outcomes, which also included an increased likelihood of patient loss during the follow-up phase [38,39,40]. Beyond the mental health burden, the detrimental impact of TB frequently contributes to the emergence of depressive symptoms, negative perceptions, behavioral disturbances, and psychosomatic manifestations [35,36].

In addition to psychosocial stressors, recent evidence suggests that individual biological vulnerability may influence how children respond to chronic infectious diseases, potentially contributing to the persistence and heterogeneity of depressive symptoms observed during tuberculosis treatment [41,42,43].

Hospitalization and isolation can have more pronounced effects on children compared to other age groups. Comparable patterns of psychological distress, coping difficulties, and the need for structured psychosocial interventions have been documented in other pediatric chronic and life-threatening conditions, including oncology, underscoring the transdiagnostic relevance of integrated mental health support [44]. They are more sensitive to social exclusion, and the effects of isolation, stigmatization, and discrimination can have long-term consequences. According to observations in the literature, the social isolation required during TB treatment has been associated with feelings of sadness, low mood, and depressive symptoms among adolescents and young adults with TB. For example, Chiang et al. reported that prolonged isolation during treatment consistently left adolescents and young adults feeling low, sad, and depressed [45]. Similarly, studies focusing on hospitalized pediatric TB patients have shown that prolonged separation from family, restricted social interaction, and isolation within hospital settings are frequently accompanied by emotional distress, depressive symptoms, and persistent negative emotional experiences [46,47]. A study conducted in Kyiv City and Cape Town reported difficulties in reintegrating into a new community, leading to social difficulties that contributed to school stigmatization [48]. Beyond TB, similar psycho-emotional and behavioral disturbances have been reported in other severe pediatric infectious diseases. Stavăr-Matei et al. demonstrated a significant association between invasive pneumococcal infections and psycho-emotional disorders, with a direct impact on both affected children and their parents, highlighting the broader psychosocial burden of serious infectious diseases in pediatric populations [49].

Several limitations should be noted. First, the retrospective design limits control for missing data and variability in documentation quality. In addition, a small proportion of patients lacked T1 assessment, which may introduce limited attrition bias; however, the proportion was low and unlikely to substantially affect prevalence estimates. Second, the screening instrument consisted of dichotomous items, which may not accurately capture gradations of severity as standardized scales do. Third, the study did not include long-term follow-up beyond T1, so the persistence of symptoms after completion of treatment could not be assessed. Despite these limitations, the study provides strong preliminary data that support the need for further prospective investigation.

## 5. Conclusions

Depressive symptoms were frequently identified among children diagnosed with tuberculosis and remained present throughout treatment. The two-stage screening approach was useful in identifying patients at potential emotional risk, while the CDI provided complementary information regarding symptom severity. Although a modest reduction in symptom burden was observed from T0 to T1, depressive manifestations persisted in a substantial proportion of patients, suggesting that emotional distress may extend beyond the initial diagnostic phase. While causal relationships and treatment outcomes were not directly assessed, these findings support the relevance of integrating mental health evaluation into routine pediatric TB care. Further prospective studies are needed to better characterize the evolution of depressive symptoms and to evaluate targeted psychosocial interventions.

## Figures and Tables

**Table 1:** Distribution of patients according to demographic and clinical characteristics.

Sociodemographic Characteristics			
Background Environment			
Rural		Urban	
134		56	
Sex			
Male	Female	Male	Female
75	59	28	28
Age			
7–10 years	29	7–10 years	11
11–14 years	36	11–14 years	20
15–18 years	69	15–18 years	25

**Table 2:** Screening outcomes for suspected depressive symptoms at baseline and follow-up.

Outcome	T0 (*n* = 190) *n* (%)	T1 (*n* = 180) *n* (%)
No suspicion of depressive symptoms	19 (10.0%)	39 (21.7%)
Suspected depressive symptoms	171 (90.0%)	141 (78.3%)

**Table 3:** Comparison of depressive symptoms at T0 and T1.

Symptom	T0 (%)	T1 (%)	*p*-Value
Affected general condition/negative mood	78.5	92.8	0.272
Problems in personal life	91.6	65.0	0.203
Ineffectiveness	49.0	52.8	0.475
Low self-esteem	73.2	68.9	0.085
Irritability	84.2	45.0	0.220
Fatigue	57.9	59.4	0.846
Insomnia	57.4	42.2	0.845
Decreased appetite	70.5	57.2	0.034
Vices/substance use	70.0	99.4	1.000
Introversion (communication issues)	70.5	76.7	0.655

**Table 4:** Pearson correlations between screening items and CDI scores at T0 and T1.

Symptom	Pearson *r* (T0-CDI)	*p*-Value (T0)	Significant	Pearson *r* (T1-CDI)	*p*-Value (T1)	Significant
Affected general condition/mood	0.129	0.076	ns	0.218	0.003	**
Problems in personal/family life	0.783	0.000	**	0.245	0.001	**
Inefficiency (work, school, etc.)	0.081	0.268	ns	0.421	0.000	**
Low self-esteem	0.194	0.007	**	0.229	0.002	**
Irritability	0.241	0.001	**	0.178	0.017	*
Fatigue	0.213	0.003	**	0.252	0.001	**
Insomnia	0.209	0.004	**	0.258	0.000	**
Decreased appetite	0.208	0.004	**	0.145	0.052	borderline
Substance use	0.203	0.005	**	0.039	0.600	ns
Introversion	0.208	0.004	**	0.188	0.011	*

Note: **—highly significant, *—statistically significant, ns = not significant.

**Table 5:** The results of the decision to perform CDI.

Decision to Perform CDI					
		Frequency	Percent	Valid Percent	Cumulative Percent
Valid	Not performed	32	16.8	16.8	16.8
	It was performed	158	83.2	83.2	100.0
	Total	190	100.0	100.0	

## Data Availability

The data supporting the findings of this study are derived from routinely collected clinical records and are not publicly available due to privacy restrictions; aggregated data are presented in this article. Further inquiries can be directed to the corresponding author.
